# Magnetic Field Patterning of Nickel Nanowire Film Realized by Printed Precursor Inks

**DOI:** 10.3390/ma12060928

**Published:** 2019-03-20

**Authors:** Chaitanya G. Mahajan, Ahmed Alfadhel, Mark Irving, Bruce E. Kahn, David A. Borkholder, Scott A. Williams, Denis Cormier

**Affiliations:** 1 Department of Industrial and Systems Engineering, Rochester Institute of Technology, Rochester, NY 14623, USA; cgm5952@rit.edu (C.G.M.); meieie@rit.edu (M.I.); 2 Microsystems Engineering, Rochester Institute of Technology, Rochester, NY 14623, USA; aha4984@gmail.com (A.A.); dabeee@rit.edu (D.A.B.); 3 AMPrint Center, Rochester Institute of Technology, Rochester, NY 14623, USA; Bruce.Kahn@rit.edu; 4 School of Chemistry and Materials Science, Rochester Institute of Technology, Rochester, NY 14623, USA; sawppr@rit.edu

**Keywords:** functional printing, metal organic decomposition, magnetic alignment, printed nickel

## Abstract

This paper demonstrates an easily prepared novel material and approach to producing aligned nickel (Ni) nanowires having unique and customizable structures on a variety of substrates for electronic and magnetic applications. This is a new approach to producing printed metallic Ni structures from precursor materials, and it provides a novel technique for nanowire formation during reduction. This homogeneous solution can be printed in ambient conditions, and it forms aligned elemental Ni nanowires over large areas upon heating in the presence of a magnetic field. The use of templates or subsequent purification are not required. This technique is very flexible, and allows the preparation of unique patterns of nanowires which provides opportunities to produce structures with enhanced anisotropic electrical and magnetic properties. An example of this is the unique fabrication of aligned nanowire grids by overlaying layers of nanowires oriented at different angles with respect to each other. The resistivity of printed and cured films was found to be as low as 560 µΩ∙cm. The saturation magnetization was measured to be 30 emu∙g^−1^, which is comparable to bulk Ni. Magnetic anisotropy was induced with an axis along the direction of the applied magnetic field, giving soft magnetic properties.

## 1. Introduction

In functional printing, metallic inks are most commonly used to produce features intended to conduct electricity (e.g., printed electronics [1]) or heat (e.g., printed heaters [2]). In recent years, researchers have begun to use a variety of different printing techniques such as Aerosol Jet^TM^, inkjet, and microextrusion to fabricate functional devices such as antennas [3,4], electrical circuit components [5,6], and sensors [7,8,9] using copper and silver metal inks. For magnetic devices, transition metals such as Fe, Co, and Ni are widely used due to their ferromagnetic properties. Despite the intense interest in printing metal-containing inks, there have been very few examples of printing ferromagnetic metals. Among these, Ni is mainly used due to its corrosion resistance, good electrical conductivity, high magnetic permeability, high thermal coefficient of resistance, and relatively high saturation magnetization [10,11]. 

The printable materials are typically formulated as inks or pastes, which require specific rheology or flow properties tuned for the selected printing process. These metal inks are commonly classified as either nanoparticle inks or precursor inks. Nanoparticle metal inks of Cu [12,13,14], Ni [15], and Ag [16,17,18] have been used in printed electronics. The stability of nanoparticle inks is affected by factors such as agglomeration of the particles and evaporation of the carrier solvent. Nanoparticle inks are prone to agglomeration over time, which can adversely affect print quality due to clogging and uneven material deposition. Evaporation of carrier solvent in the nanoparticle-based inks, during printing with some aerosol-based printing processes, leads to an increase in the ink’s solid loading content, thus altering the ink’s rheological properties [19]. 

Precursor inks (which are also known as metal organic decomposition or MOD inks) have received considerable interest in the research community due to their potential for overcoming some of the challenges with nanoparticle inks. Precursor inks are formulated by dissolving an organic metal complex in an appropriate solvent to produce a homogeneous solution. These inks do not contain metal particles and do not suffer from some of the particle instability issues of particle-based inks. However, the typical metal content of precursor inks is significantly lower than that of nanoparticle inks. For instance, Rosen et al. formulated a copper precursor ink with a loading of 5.2 wt % [20]. This is compared with a copper nanoparticle gravure ink formulated by Fan et al. with a loading of 35.15 wt % [21]. Precursor inks of different metals such as Cu [22,23,24,25], Au [26] and Ag [27,28,29,30] have been formulated and used in printed electronic applications. During reduction from its complexes, Ni usually oxidizes upon contact with air. This has made Ni precursor ink formulation challenging in the past. 

Ni nanoparticles and nanowires have been formulated with different techniques, such as hydrothermal processing [10,31,32], chemical reduction [33,34,35], and electrochemical deposition [36]. However, the methods to fabricate, deposit, and/or align the nanowires onto desired substrates typically requires multiple steps, large quantities of electrically or functionally inactive materials (templates), and extreme conditions. For example, the reduction of Ni to its elemental state can be achieved in a stainless steel autoclave, and must be followed by washing and drying of the nanowires in an inert atmosphere to avoid oxidation [10,31,33,35]. 

Deposited nanowires containing Ni have many applications, including surface-enhanced Raman spectroscopy (SERS) [37,38], magnetic data storage [39,40] and giant magnetoresistive sensors [41,42,43,44]. These magnetic applications exploit the fact that the large shape anisotropy generated from the high aspect ratio of nanowires provides high intrinsic coercivity. Aligned nanowires on surfaces can also be useful for optical applications, such as nanowire polarizers [45,46], and for many biological purposes, for example, “lab on a chip” devices, as well as for the construction of tubular sensors which exploit geometrically induced circumferential magnetization [47], or for cell guidance using tissue- or organ-like structures in vitro [48]. There is, therefore, significant interest in developing a method to directly fabricate nanowires having the desired orientation onto the preferred substrate. 

Various printing techniques have been developed to pattern metal inks onto the desired substrate [49]. Typical examples include flexography, gravure, screen printing, inkjet, and aerosol printing. Among these processes, inkjet and aerosol printing are capable of on-demand material printing. They are able to print using very small quantities of ink, and they are non-contact printing processes. In an Aerosol Jet^TM^ system, the ink is either pneumatically or ultrasonically atomized into an aerosol that is focused in a nozzle, and directed towards the substrate. Larger standoff distances are possible with aerosol than with inkjet printing. This feature enables printing of the ink on planar as well as non-planar substrates. Aerosol Jet^TM^ printing has been used to fabricate different functional devices such as transistors [6], sensors [8,9], and strain gauges [9]. 

**Figure 1:**
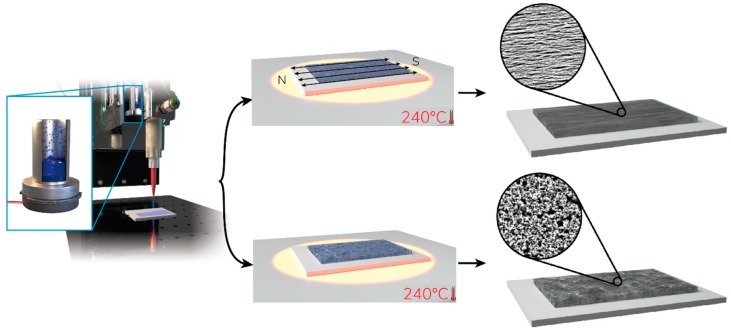
Schematic illustration of printing a Ni precursor ink and sintering it in presence of homogeneous magnetic field to reduce the complex to aligned nanowires.

In this work, a Ni precursor ink was synthesized in ambient conditions such that the ink can be thermally reduced to elemental nickel after printing. This homogeneous ink can be formulated for many different printing and deposition processes. The ink was printed on different substrates using an aerosol printing technique. The reduction of Ni was observed in the presence and absence of a magnetic field. Interestingly, reduction in the presence of a magnetic field produced pure, template-free, aligned Ni nanowires. In this way, large areas of aligned Ni nanowires were produced, using only weak magnetic fields. We believe this novel method is the first example of aligned nanowire formation on a surface by a thermal reduction (curing/sintering) process. Moreover, the reaction is simple and the byproducts are volatile, leaving quantitatively pure Ni nanowires, without the need for further purification. The electrical and magnetic properties were enhanced in the direction of the aligned Ni nanowires. The film morphology can be easily manipulated during the reduction process to produce a number of different novel structures having unique electronic and magnetic properties. Figure 1 shows the schematic illustration of printing a Ni precursor ink and reducing it in the presence of homogeneous magnetic field to produce aligned nanowires.

## 2. Materials and Methods

### 2.1. Materials

All the chemicals used were analytical grade. Nickel formate (molecular weight (MW): 148.74) was purchased from Alfa Aesar, Ward Hill, MA, USA, and ethylene diamine was purchased from Fisher Scientific, Fair Lawn, NJ, USA These chemicals were used without further purification. Glass microscope slides (Thermo Scientific, Waltham, MA, USA) were used as substrates and were cleaned with isopropyl alcohol (IPA) before printing.

### 2.2. Ink Formulation

Ethylenediamine (0.72 mL, 10.77 mmol) was dissolved in 4 mL of distilled water. Nickel formate (0.8 g, 5.38 mmol) was added to this solvent mixture and stirred at room temperature for 15 min. The ink was filtered through a 0.2 µm syringe filter. Other additives, such as 1% (w/w) of polyvinylpyrrolidone (MW: 3500, K12) and 20 µL of BYK 333 surfactant (10% (v/v) solution in distilled water) were added to the ink. 

### 2.3. Aerosol Printing

To dispense the Ni ink on glass slides, a NanoJet aerosol printer (Integrated Dispensing Solutions, Inc., Albuquerque, NM, USA) was used. The NanoJet printer uses ultrasonic energy to atomize the functional ink. A carrier gas delivers the aerosol to a series of aerodynamic focusing lenses that concentrate the aerosol as it exits the nozzle. The ultrasonic atomizer consists of a planar piezoelectric transducer which is acoustically coupled with the ink at frequencies in the range of 1 to 2 MHz. The atomizer produces a polydisperse distribution of droplets with a size distribution in the range of ~0.5 to 5 µm in diameter [50]. Air was used as a carrier gas to transport the aerosol to the focusing lenses. Ink passes through the focusing lens and into a tapered luer lock dispensing tip. A sheath gas flow was used to avoid clogging of the nozzle and to focus the distribution of aerosol droplets onto the substrate. The sheath gas and aerosol flow rate were kept constant throughout the experiments to maintain consistency in the printed samples. The printer uses Aerotech PRO 165 mechanical-bearing linear stages to move the work table in the X and Y directions, and an Aerotech PRO 115 mechanical-bearing linear stage to move the deposition head in the Z direction. A solid 1 cm × 1 cm square pattern was printed using a 25 gauge dispensing tip. The distance between the substrate and nozzle tip was kept constant at 5 mm, and a translational speed of 2 mm/s was used to print the samples. The printed pattern had a wet film thickness of ~4 µm for a single printed layer. 

### 2.4. Ink Characterization

The surface tension of Ni ink was measured using a contact angle goniometer and tensiometer (Model 250, ramé-hart, Succasunna, NJ, USA) using the pendant drop method. The viscosity of the ink was measured using a microVISC viscometer (RheoSense, San Ramon, CA, USA). The surface tension and viscosity of the ink were 44.01 mN/m and 3.2 cP, respectively. To improve the wettability of the ink, the substrates were treated with atmospheric plasma (Surfx Atomflo, Redondo Beach, CA, USA). Thermogravimetric analysis (TGA) and differential thermal analysis (DTA) (Shimadzu DTG-60, Kyoto, Japan) were performed by heating the sample in an aluminum pan from room temperature to 400 °C at 10 °C/min. Surface morphology and elemental analysis of the printed films were studied using a scanning electron microscope with energy-dispersive X-ray spectroscopy (EDS) (Jeol, JSM- IT100LA, Peabody, MA, USA). The thickness of the printed films was measured using an optical profilometer (Nanovea ST400, Irvine, CA, USA) and a stylus profilometer (Tencor P2, Milpitas, CA, USA). Electrical conductivity was measured using a four-point probe (Jandel RM3000, Leighton Buzzard, UK). Magnetic properties were studied by obtaining the hysteresis loops using a Princeton Applied Research (PAR 155) vibrating sample magnetometer (VSM) modified with Lake Shore Cryotronics 7300 electronics (Westerville, OH, USA).

### 2.5. Alignment Characterization

In order to quantitatively evaluate the orientation of reduced Ni nanowires, a method used by Ayres et al. [51] to measure the fiber alignment in electrospun materials was adopted. 2D fast Fourier transform (FFT) was used to quantify the orientation of nickel nanowires in each image. Each SEM image was first cropped to a size of 512 × 512 pixels. The cropped images were then converted into grayscale images. FFT analysis was performed on each grayscale image using the oval profile plug-in in the ImageJ software package. The plug-in approximates each nanowire in a given image as a long slender oval using image processing algorithms. The FFT computed by ImageJ maps the orientation of the major axis of each oval into a nanowire orientation frequency domain. The peak shape and height in the 2D FFT plot determine the degree of nanowire alignment, while the peak position indicates the axis of orientation of the nanowires. 

## 3. Results and Discussion

### 3.1. Ni Ink Characterization

The Ni ink contains nickel formate complexed with ethylenediamine, which undergoes a thermal reduction process to generate metallic Ni on the desired substrate following printing and curing. The use of formate counter ions decreases the mass of the organic content of the complex, provides a relatively low decomposition temperature, and subsequently decreases the residue following decomposition [52]. Furthermore, the decomposition of nickel formate is accompanied by the release of carbon oxides and molecular hydrogen [53], which contribute to the reducing atmosphere, thus preventing the oxidation of Ni. The bidentate ethylenediamine has been used in the formation of the metal complex to enhance the reduction efficiency, achieve complex stability, and increase complex solubility in water [27]. 

**Figure 2:**
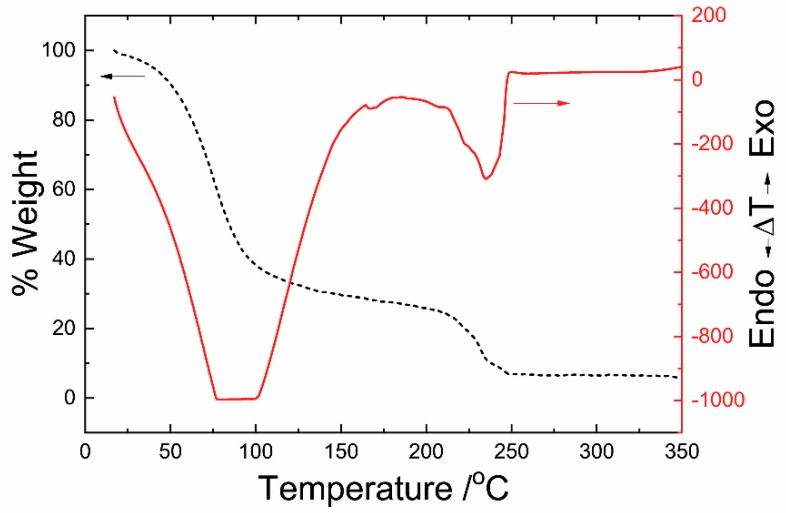
Thermogravimetric analysis (TGA) and differential thermal analysis (DTA) of the Ni metal organic decomposition (MOD) ink.

The TGA of the Ni ink shows that the thermal decomposition occurs in two stages (Figure 2, dashed black line). The first stage includes the solvent (water) evaporation up to 100 °C, and the second stage involves the reduction of Ni to its elemental state at 235 °C. The final amount of Ni in the ink was 5.7% (w/w). According to DTA data (Figure 2, solid red line), the first endothermic peak was observed around 80 °C with the corresponding mass loss indicating solvent evaporation, while the last endothermic peak was observed around 235 °C, indicating the reduction of Ni to its elemental state. This temperature is the minimum necessary to reduce the ink to metallic Ni. For this reason, 240 °C was chosen as the curing temperature for subsequent processing. It is important to clarify that the metal formation process of these inks is mechanistically very different from that of conventional (nano)particle conductive inks. The initial step of curing these inks is a chemical (reduction) process, rather than a physical process (as is observed in particle-based inks). As such, the curing temperature is chosen based upon careful analysis and characterization of the chemical reduction process of Ni upon heating. 

**Figure 3:**
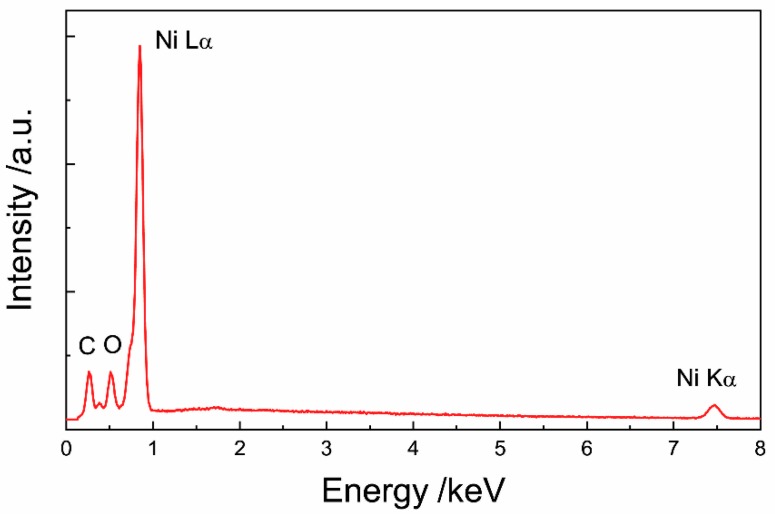
EDS spectrum of printed and sintered Ni MOD ink.

The EDS spectrum of reduced Ni ink (Figure 3) shows peaks for Ni at 0.851 and 7.471 keV. A small amount of carbon and oxygen was also detected in the EDS spectrum, which was either the byproduct of organic decomposition or contamination. 

### 3.2. Printing of Ni Ink

**Figure 4:**
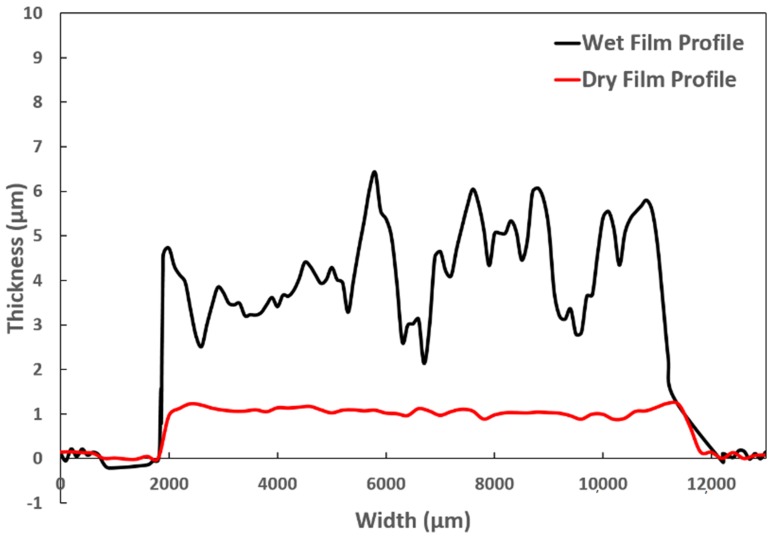
Optical profile for wet film and dry film for a single layer.

To measure the electrical and magnetic performance of the ink, the NanoJet aerosol printer was used to dispense the Ni ink onto the substrate. Other printing processes, such as inkjet printing, can also be used to deposit this ink [27]. Glass slides were used as substrates and were cleaned with isopropyl alcohol before printing. To improve the wettability of the ink, the substrates were treated with atmospheric plasma. Figure 4 shows the optical profile for the wet and dry film thickness for one layer. The change in thickness during drying and curing is due to evaporation of the ink carrier liquid. Based upon the results from the DTA discussed above, the final printed samples were cured at 240 °C for 15 min. Two different cases were explored to study the sintering behavior. In Case 1, the samples were sintered at 240 °C for 15 min in the absence of any magnetic field. In Case 2, the samples were sintered at 240 °C for 15 min in a homogeneous magnetic field of 250 Oe. 

### 3.3. Reduction of Ni Ink

**Figure 5:**
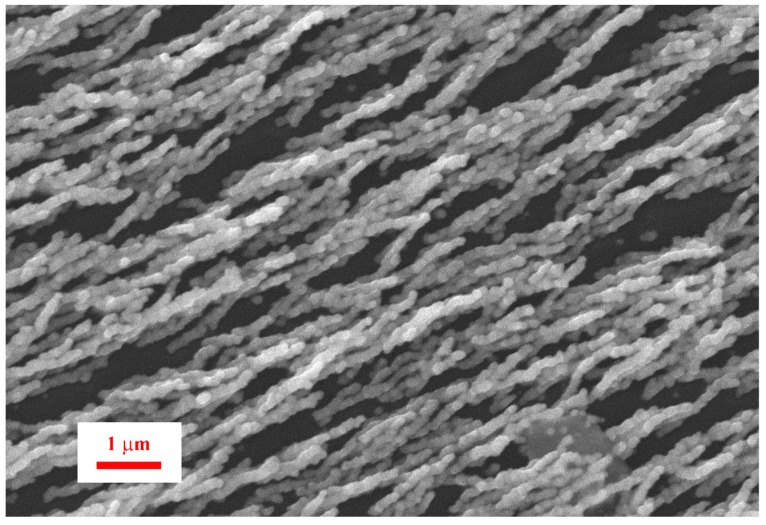
SEM image of aligned Ni nanowires (50% dilution).

**Figure 6:**
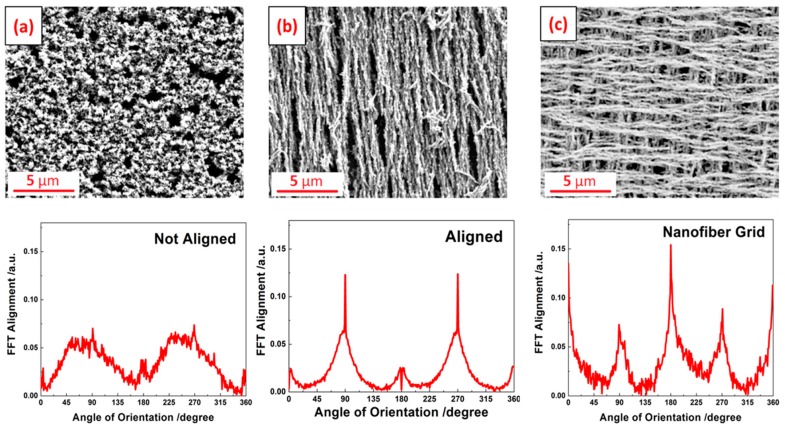
SEM images with corresponding 2D FFT alignment plots of two nickel layers sintered in (**a**) no magnetic field, (**b**) homogeneous magnetic field of 250 Oe in the same direction for all layers, and (**c**) multilayer printing and heating in presence of homogeneous magnetic field with alternating 0° and 90° orientations between layers.

After aerosol printing of the samples, the glass substrate was heated on a benchtop hot plate. The cured Ni films were characterized by SEM imaging (Figure 5 and Figure 6) to study the morphology and structure of the reduced Ni. The samples that were processed in the presence of a homogeneous magnetic field showed nanowires where the nanowire axis was aligned in the direction of the magnetic field. The SEM shows that each nanowire is composed of individual 100–250 nm nanoparticles that are fused together, resembling a “string of pearls”. This suggests that the reduction forms nanoparticles which are aligned in the presence of the magnetic field. Those aligned nanoparticles then sinter together at the processing temperature. The resulting Ni nanowires follow the pattern of the magnetic field lines.

In the absence of a magnetic field, the particles are not aligned to form nanowires. Rather, the samples that were thermally processed in the absence of a magnetic field show the presence of a porous Ni film with particles ranging from 100 to 250 nm (Figure 6a). Significant necking between Ni particles was observed. The samples were cured after each printed layer. For printing of the second and third layers, the Ni ink was printed on top of the previously cured layer. In order to study the isotropy of the printed Ni, an extra case was investigated where a nanowire grid was printed by reducing the Ni in the presence of a magnetic field perpendicular to the previous layer (Figure 6c). 

The FFT alignment plot for Ni cured in the presence of magnetic field show a fibrous structure with peaks at 90 and 270°, while the Ni reduced in absence of magnetic field shows random particulate structure. The nanowire grid sample produced using alternating magnetic field orientations shows peaks at 0, 90, 180, 270, and 360°. The intensities of the peaks at 0, 180, and 360° were higher than that of 90 and 270° because the alignment of the top layer was more prominent in the SEM image than that of the bottom layer.

### 3.4. Electrical Characterization

**Figure 7:**
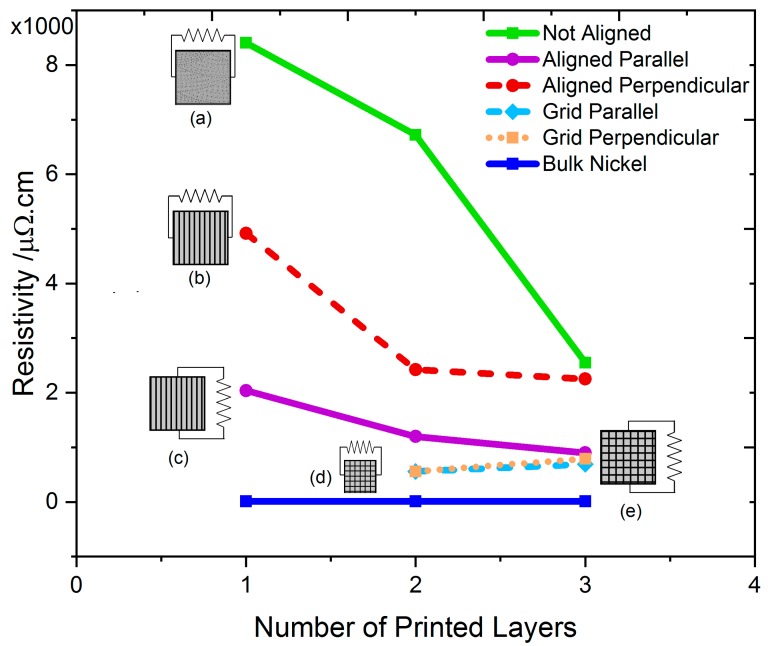
Electrical characterization of Ni for particles (**a**) not aligned, (**b**,**c**) aligned (nanowires), and (**d**,**e**) in grid orientation

The electrical resistivity for the printed Ni ink was measured using a four-point probe. A significant difference in the electrical properties was observed for Ni cured in the presence of a magnetic field compared with that of Ni cured in the absence of a magnetic field. Figure 7 shows the resistivity values for the different cases. As expected, the resistivity decreased as the number of layers increased. The reason for the decrease was that the voids generated during the sintering of the previous layer were filled with additional Ni during the printing of subsequent layers. The electrical resistivity of the Ni cured in the presence of the magnetic field was lower (higher conductivity) than the Ni cured in the absence of a magnetic field. The resistivity was lower in the direction of the aligned Ni nanowires than perpendicular to the aligned Ni nanowires. For the nanowire grid samples produced using alternating magnetic field orientations, the resistivity was almost equal in both directions. The lowest resistivity of 560 µΩ∙cm (80X bulk Ni) was observed in the nanowire grid samples, which is better than previously published studies [54,55]. It should be noted that these nanowire arrays are not completely dense (there are spaces between nanowires). These resistivities are calculated assuming uniform coverage of Ni (no pores), so they overestimate the actual resistivity based upon the amount of Ni actually present in the nanowires.

### 3.5. Magnetic Characterization

**Figure 8:**
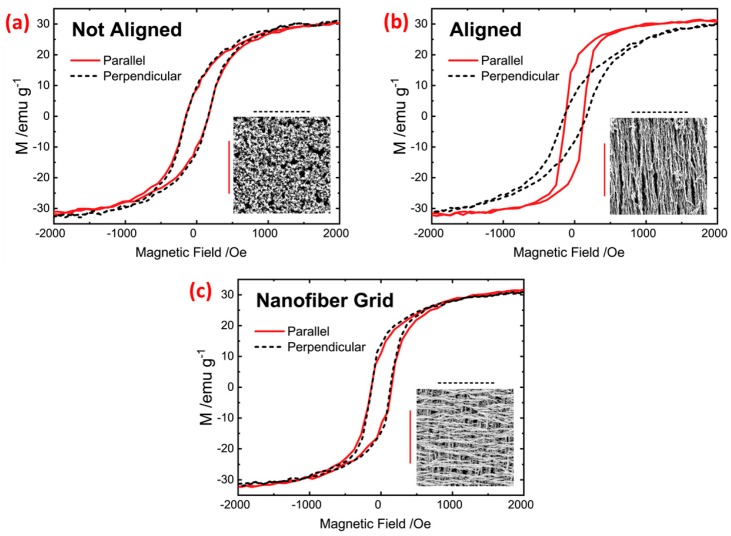
Magnetic characterization of Ni for particles (**a**) not aligned, (**b**) aligned (nanowires), and (**c**) in grid orientation.

The magnetic properties of two-layer Ni films reduced in the absence of a magnetic field (Figure 8a) and films reduced in the presence of a directional magnetic field (Figure 8b) were studied by measuring the hysteresis loops. The saturation magnetization for all samples was found to be 30 emu∙g^-1^, which is comparable to the bulk Ni saturation magnetization [56]. Figure 8a shows hysteresis loops obtained in planar perpendicular directions where soft magnetic properties were observed with remanent magnetization of 10 emu∙g^-1^ and coercive field of 120 Oe. By exposing the film to a directional magnetic field during the reduction process, a significant difference was obtained. The induced anisotropy parallel to the alignment direction showed a remanent magnetization of 20 emu∙g^−1^, while the perpendicular direction shows a remanent magnetization of 7 emu∙g^-1^. This is due to the obtained nanowire structure having a shape anisotropy along the axis of the nanowire. By aligning the film layers in perpendicular directions to form a nanowire grid structure (Figure 8c), higher remanent magnetization than the unaligned film was obtained in the parallel and perpendicular directions due to the connected nanowire grid structure. These results show the possibility of tailoring the magnetic properties of the printed Ni films, which can be exploited for applications such as magnetic data storage or magnetoresistive sensors. The nanogrid structure on a wide range of substrates can also be explored for making skin-attachable loudspeakers and microphones [57]. 

## 4. Conclusions

A novel template-free method of producing aligned Ni nanowires in ambient conditions from a homogeneous solution was presented. A Ni precursor (MOD) ink was synthesized, printed, cured, and used for electronic and magnetic applications. This was one of the few examples of printing ferromagnetic inks. The ink can be printed using a variety of processes on different substrates, including flexible and thermally sensitive materials for applications such as antennas, magnetic sensors, and optical polarizers. Aerosol printing of the ink on glass substrates was demonstrated in this study. Inks with different rheological properties can also be easily developed for other functional printing techniques, such as flexography, inkjet printing, and screen printing. Thermal reduction of the precursor ink in the presence of a weak magnetic field produced large areas of pure aligned Ni nanowires, requiring no further processing, which have enhanced anisotropic electrical and magnetic properties. This was one of the first examples of printed metallic Ni from a precursor, and the first example of thermally induced nanowire formation on a substrate. The film structure and morphology can be easily manipulated, enabling the production of a variety of novel patterned structures having unique electronic and magnetic properties, as well as commercial applications. The lowest electrical resistivity (highest conductivity) was observed for nanowire grids. Further research is planned to reduce the electrical resistivity and to study the Ni alignment using other (for example photonic) sintering techniques.
